# Geo-analysis: the distribution of community health workers in relation to the HIV prevalence in KwaZulu-Natal province, South Africa

**DOI:** 10.1186/s12913-022-07707-x

**Published:** 2022-03-11

**Authors:** G. E. Khumalo, S. Ntuli, E. Lutge, T. P. Mashamba-Thompson

**Affiliations:** 1 University of KwaZulu-Natal, Discipline of Public Health Medicine, School of Nursing & Public Health, Howard College Campus, Durban, South Africa; 2 Health Research & Knowledge Management Unit; KwaZulu-Natal Department of Health, 330 Langalibalele Street, Pietermaritzburg, South Africa; 3 Geographical Information System Unit, African Health Research Institute, Somkhele, Mtubatuba, South Africa; 4 Faculty of Health Sciences, University of Pretoria, Prinshof Campus, Bophelo Rd, Prinshof 349-Jr, Pretoria, 0084a South Africa

**Keywords:** HIV prevalence, Community health worker distribution, Geospatial, KwaZulu-Natal

## Abstract

**Background:**

The South African Ward Based Primary Health Care Outreach Team (WBPHCOT) policy framework states that the distribution of community health workers (CHWs) should be proportional to levels of poverty and disease within the population. We aimed to describe the spatial distribution of CHWs in relation to the prevalence of the Human Immunodeficiency Virus (HIV) which has itself been associated with poverty in previous studies.

**Methods:**

This was a descriptive, cross-sectional study in which secondary data was used for geospatial analysis. Based on the extrapolation from the norm of one WBPHCOT per 6000 individuals, we utilized geographic information system (GIS) methods to visualize the distribution of CHWs in relation to the prevalence of HIV in KwaZulu-Natal (KZN). Dot density mapping was used to visualize the random distribution of CHWs in relation to HIV prevalence and population in the districts. The districts’ HIV prevalence, number of PLWH, ratio of CHW: people living with HIV (PLWH), ratio of CHW: population and poverty scores were mapped using choropleth mapping. MapInfo Pro 17.0 was used to map geospatial presentation of the data.

**Results:**

Overall, KZN province showed under allocation of CHWs with a CHW: people ratio of 1: 1156 compared to the estimated norm of 1: 600–1000. At district level, only two of 11 districts met the suggested norm of CHW: PLWH (1: 109–181). This indicates shortages and misallocation of CHWs in the nine remaining districts. Furthermore, our findings showed extensive geospatial heterogeneity with no clear pattern in the distribution of CHWs. There was no relationship between CHW distribution and HIV prevalence or poverty scores in the districts.

**Conclusion:**

This study shows inequality in the distribution of CHWs which may be associated with inequalities in the provision of HIV related services. It is critical to strengthen the response to the HIV epidemic through the appropriate distribution of CHWs especially in those districts with high levels of HIV prevalence and poverty.

## Background

KwaZulu-Natal (KZN) province has the highest prevalence of Human Immunodeficiency Virus (HIV) among adults in South Africa (at 18.1%), [1] as well as the highest HIV prevalence among pregnant women (of 41.1%) [2]. According to Statistics South Africa (Stats SA), there are just over two million people living with HIV (PLWH) [3].

Previous studies have shown that a relationship exists between HIV and poverty [4–11]. This complex association has been demonstrated in South African and globally [6, 8, 9, 11]. Of the total population of 11,065,240 million in KwaZulu-Natal [12], 3.2 million people live in conditions of extreme poverty [13]. KwaZulu-Natal is the third poorest province in South Africa, with communities in the rural areas having particularly high levels of poverty [14, 15]; these have been associated with high HIV prevalence [16–19].

Despite considerable progress made by the KZN province in addressing the HIV epidemic, the HIV incidence remains high especially among adolescent girls and young women [20]. Community health workers (CHWs) have been shown to be effective in mitigating the HIV pandemic through their provision of chronic disease support; they are also increasingly recognized as a cost-effective cadre of health worker which mitigates staff-shortages in different settings [21]. In KZN, CHWs should be deployed preferentially to impoverished communities, where access to health care facilities has been shown to be poor [14, 22–24]. They are to first serve communities living on less than Statistics South Africa’s upper bound poverty line of R1183 per person per month, calculated on the cost of basic needs [23, 25].

However, due to shortages of CHWs in KZN, some communities do not receive the HIV services offered by them [22] and hence miss an opportunity to be linked to healthcare [26, 27].

As part of the re-engineering of primary healthcare (PHC) in 2010, the South African Department of Health (NDoH) launched a Ward-Based Primary Health Care Outreach Team (WBPHCOT) policy framework [23]. It is on this policy framework that the study is based and from which the CHW distribution ratios are extrapolated. The WBPHCOT is supervised by an outreach team leader (OTL) who is usually a nurse and the CHWs are key components of the team.

The roles of CHWs have been expanded as a strategy to strengthen HIV healthcare services and provide longitudinal patient support in KZN [21, 28, 29]. The HIV services that CHWs provide include patient support (counselling, home-based care, education, adherence support and livelihood support) and health service support (screening, referral and health service organization and surveillance) [21, 28–30]. Community HIV interventions by CHWs have been seen as an important predictor of HIV treatment success [31]. The community HIV interventions include linking PLWH to HIV healthcare, promoting ART adherence, and improving retention in care [21, 32–34].

The distribution of CHWs is influenced by community poverty, distance and travel time between households, demographic characteristics of the population and the burden of disease in the particular population [23]. This is in line with the suggestion of the World Health Organization (WHO), that understanding the local geography and the disease epidemiology will assist in optimizing the CHW programme [35, 36]. This can be achieved using geospatial analysis. In HIV related research, geospatial analysis has been successfully used, for example, to map male medical circumcisions [37], determine areas of high HIV prevalence [38], and determine spatial relationships between HIV prevalence and social covariates [10]. Therefore, using geospatial analysis, our study aimed to map and describe the distribution of CHWs in relation to the HIV prevalence in the districts where they are deployed. The objectives of the study were to describe CHW distribution in relation to the district population, HIV prevalence and poverty scores in the districts.

The study findings will therefore illuminate shortfalls and strengths in the allocation of CHWs in relation to the HIV prevalence in each KZN district and highlight areas of need.

## Study methods

### Study setting

**Fig. 1:**
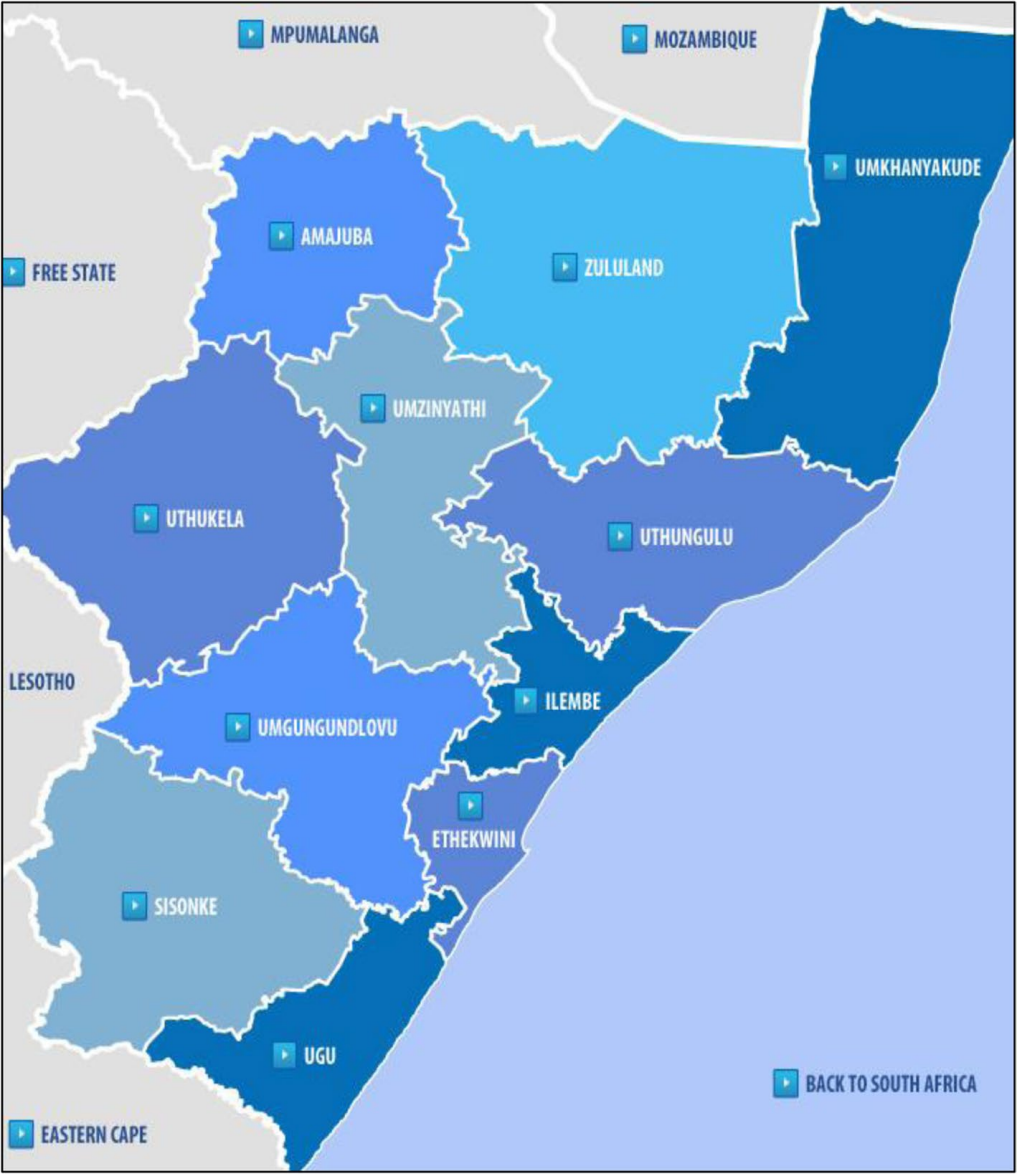
Map of KwaZulu-Natal Province showing 11 districts [Source: Eyethu KZN]

The study was conducted in KZN, the province with the second largest population in SA. KwaZulu-Natal has a population of 11,065,240 million which translates to 19.7% of the South African population [12, 39]. As seen in Fig. 1., KZN comprises 11 districts, namely Ugu, uMgungundlovu, uThukela, uMkhanyakude, King Cetshwayo, Harry Gwala, uMzinyathi, Amajuba, Zululand, iLembe and eThekwini (which is classified as a metropolitan municipality). While eThekwini is the smallest in terms of land area size, it is the most populous district in the province [3].

Zululand covers the largest proportion of land area in the province, spanning approximately 14,800 km^2^. It accounts for about 15,7% of the total land area of KZN [12] as seen in Table 1.

**Table 1:** District population density and land size in the KwaZulu-Natal districts

District	Size of district (km^2^)^a^	Proportion of KZN land area (%)^a^	Population density (persons per km^2^) ^a^	Degree of urbanization^b^
uMkhanyakude	13,855	14.68	50	rural
Zululand	14,799	15.68	60	rural
uThukela	11,134	11.79	63	semi-urban
Amajuba	7102	7.52	75	urban
uMzinyathi	8652	9.16	64	rural
uMgungundlovu	9602	10.17	114	semi-urban
iLembe	3269	3.46	201	semi-urban
King Cetshwayo	8213	8.70	118	urban
eThekwini	2556	2.70	1448	semi-urban
Harry Gwala	10,386	11.00	49	rural
Ugu	4791	5.07	157	urban

^a^KwaZulu-Natal Community Survey, 2016

^b^KwaZulu-Natal district profiles (https://www.cogta.gov.za/ddm/index.php/2020/07/06/kwazulu-natal-profiles/)

The uMgungundlovu District Municipality is home to Pietermaritzburg, the capital city of KZN [39]. Table 1. tables the population density and land size of the districts and as well as the degree of urbanization for each district. As seen in Table 1., KZN has a mixture of rural, urban and semi-urban districts.

### Study design

The study was a descriptive, cross sectional study. We use secondary data from different sources mentioned below to conduct geospatial analysis.

### Data sources

To describe the distribution of CHWs in the province in relation to HIV prevalence for each district, the number of CHWs allocated to each district was obtained from the Human Resource Department of the KZN Department of Health (KZN-DoH) in 2019. The CHWs were all employed and paid a stipend by the KZN-DoH. The CHW information can only be obtained from an authorized user at KZN-DoH, HR department. It is obtained as an anonymized excel spreadsheet with numbers of staff categories in each clinic.

For the KZN HIV prevalence, we used data from The South African National HIV Prevalence, Incidence, Behaviour and Communication Survey [1]. The South African National HIV Prevalence, Incidence, Behaviour and Communication Survey is a cross-sectional, population-based, household survey conducted using multi-stage stratified cluster random sampling; it is currently used to estimate the official national and provincial prevalence of HIV in South Africa [1]. The last one was conducted in 2017 when the KZN HIV prevalence was recorded as 18.1% [1], which is the rate used in this study.

The district HIV prevalence data was obtained from the 2017 South African, National Antenatal Sentinel HIV Survey Report which describes antenatal HIV prevalence in pregnant women between the ages of 15–49 years [2].

The number of PLWH in the districts was estimated from the number who tested positive for HIV in 2017 obtained from the KZN-DoH District Health Information System (DHIS). This is a database that houses health related data used for planning and management of health service delivery [40] .

The KZN district shapefiles, which store digital information such as the geometric location of the districts, were obtained from the Geographical Information System (GIS) Unit of KZN-DoH.

Poverty data was obtained from the South African Multidimensional Poverty Index (SAMPI) collected and reported by Stats SA [41] . The SAMPI, is based on the Alkire-Foster method and provides another powerful tool in Stats SA’s ongoing efforts to measure poverty and deprivation in the country [41]. The strength of this index using census data rests in the ability to reliably map poverty down to the specific geographical level required [41]. The SAMPI is an indicator used to measure poverty and is made up of several factors which include poor health, lack of education, inadequate living standards, lack of income, disempowerment, lack of decent work and threat from violence [41, 42]. In this study, SAMPI will be referred to as the Multidimensional Poverty Index (MPI).

### Estimating the CHW: PLWH ratio

We estimated the CHW: PLWH ratio as an extrapolation from the norm of one WBPHCOT per 6000 individuals [23] and the 18.1% HIV prevalence for KZN [1]. Considering the 18.1% HIV prevalence in the province [1], we estimate that one WBPHCOT serves 1086 PLWH. Each WBPHCOT should include between 6 and 10 CHWs [23] which implies that one CHW should visit between 109 and 181 PLWH, that is, a ratio of 1:109–181.

We estimated the number of PLWH based on the total number of people that had tested positive for HIV in the districts by 2018. This data is available on the KZN-DoH DHIS [40]. Statistics South Africa (Stats SA) reported that there are 2,133,000 million PLWH in KZN [3] whereas in the DHIS, the total number of people that tested positive for HIV were 2,775,039. In order to err on the side of over-estimating rather than under-estimating the number of PLWH in KZN, we used DHIS data to estimate the number of PLWH in the districts.

### Geospatial analysis

Geospatial analysis, using MapInfo Pro 17.0, was used to create visualizations of the distribution of CHWs in relation to the HIV prevalence, number of PLWH, district populations, CHW: PLWH ratio’s and poverty scores. Geospatial analysis is a GIS based approach used to analyze geographical referenced information using methods such as statistics, information theories, and geo-visualization techniques [43]. Dot density mapping, where one dot represented 32 units, was used to visualize the random distribution of CHWs in relation to HIV prevalence in the districts. The districts’ HIV prevalence, number of PLWH, population, CHW: PLWH ratio and poverty scores were mapped using choropleth mapping, with intense and light colours representing the relevant data in each map. Shapefiles obtained from the GIS Unit of the KZN-DoH, were used to define the geographical locations of districts, district population, CHWs and PLWH. MapInfo Pro 17.0 conducts built-in thematic layering equations, scoring mechanism and analysis of the data imported to create univariate or bivariate thematic maps that superimpose two variables, for example CHW distribution and HIV prevalence.

## Results

### Community health workers distribution per district population

According to the Human Resource (HR) department of KZN Department of Health (KZN-DoH), the province has 9573 CHWs and 557 WBPHCOTs (HR, KZN-DoH, March 2019). The norm as per the WBPHCOT policy framework, requires that each CHWs serve between 600 and 1000 people, a ratio of 1:600–1000. As seen in Table 2., KZN province has a smaller ratio of 1: 1156 indicating an inappropriate distribution and a shortage of CHWs in the province. The CHW: people ratio ranged from 1: 630 (Harry Gwala district) to 1: 2370 (eThekwini district).

**Table 2:** The general distribution of CHWs in relation to district population in KwaZulu-Natal

	Province/District	District population^a^	Number of CHWs^b^	CHW: People ratio Norm = 1CHW: 600–1000 people
	KwaZulu-Natal	11,065,240	9573	1: 1156
Within the norm	Harry Gwala	510,865	811	1: 630
	iLembe	657,612	941	1: 699
	uMkhanyakude	689,090	817	1: 843
	Zululand	892,310	1008	1: 885
	uThukela	706,588	787	1: 898
	Ugu	753,336	824	1: 914
	King Cetshwayo	971,135	1035	1: 938
Out of the norm	Amajuba	531,327	485	1: 1096
	uMzinyathi	554,882	495	1: 1121
	uMgungundlovu	1,095,865	808	1: 1356
	eThekwini	3,702,231	1562	1: 2370

^a^[Source: KwaZulu-Natal Community Survey, 2016 (Statistics, South Africa]

^b^[Source: KwaZulu-Natal Department of Health, Human Resource Electronic Persal system, 2019]

**Fig. 2:**
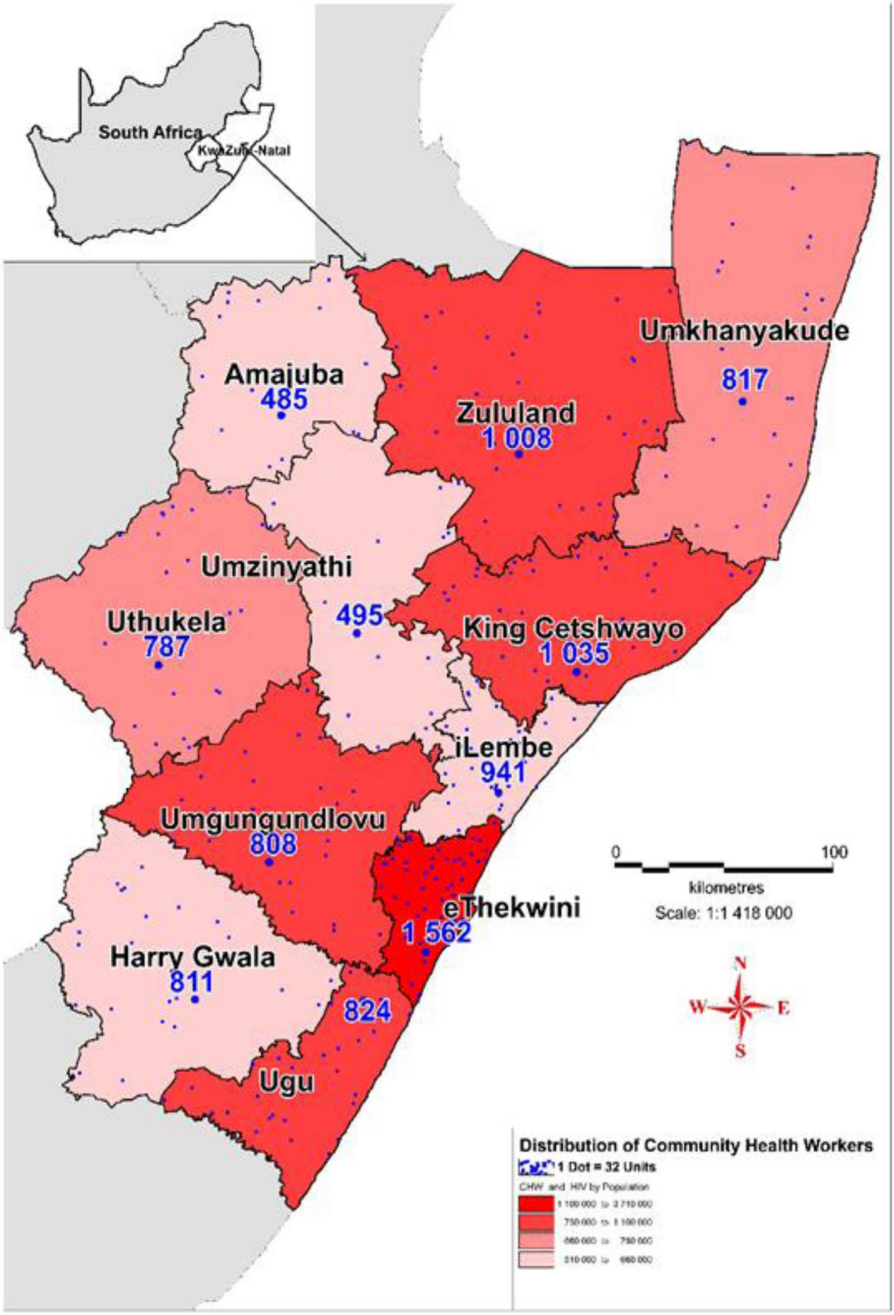
Bivariate choropleth map showing spatial distribution of CHWs (dots) in relation to district populations in KwaZulu-Natal districts. The high intensity colours representing the most populous districts

Of the 11 districts, seven (64%) met the distribution norm of CHW: people ratio and four (36%) did not. Although the majority of the districts showed sufficient numbers of CHWs, there was no clear pattern in the allocation of CHWs in general. The two districts with the highest population, that is, eThekwini and uMgungundlovu, show the greatest shortage of CHWs with ratios of 1:2370 and 1: 1356 respectively. As seen in Fig. 2, eThekwini has the highest population and has the greatest shortage of CHWs. The other example of the unexplainable misallocation of CHWs in the districts, is seen in the example of uMgungundlovu and Harry Gwala districts. As seen in Fig. 2., uMgungundlovu district has almost twice the population of Harry Gwala district. However, uMgungundlovu district has almost the same number of CHWs as Harry Gwala district (808 versus 811).

As seen in Fig. 2., the district with the lowest population, Harry Gwala, had the highest CHW: people ratio of 1:630 which met the norm suggested by the WBPHCOT policy framework. However, Amajuba district, which has the second lowest population, had one of the lowest CHW: people ratio of 1: 1096, that did not meet the suggested norm. The district with second highest CHW: people ratio of 1:699 was iLembe district. However, iLembe is one the districts with the highest populations. The other distinct misallocation of CHWs is noted when comparing King Cetshwayo and uMgungundlovu districts. King Cetshwayo district has a smaller population compared to uMgungundlovu district but was allocated more CHWs than uMgungundlovu. Overall, the majority of the districts showed an appropriate distribution of CHWs and hence sufficient CHWs. However, the distribution of CHWs in the districts did not follow an obvious pattern.

### Community health workers distribution, HIV prevalence and CHW: PLWH ratio

**Fig. 3:**
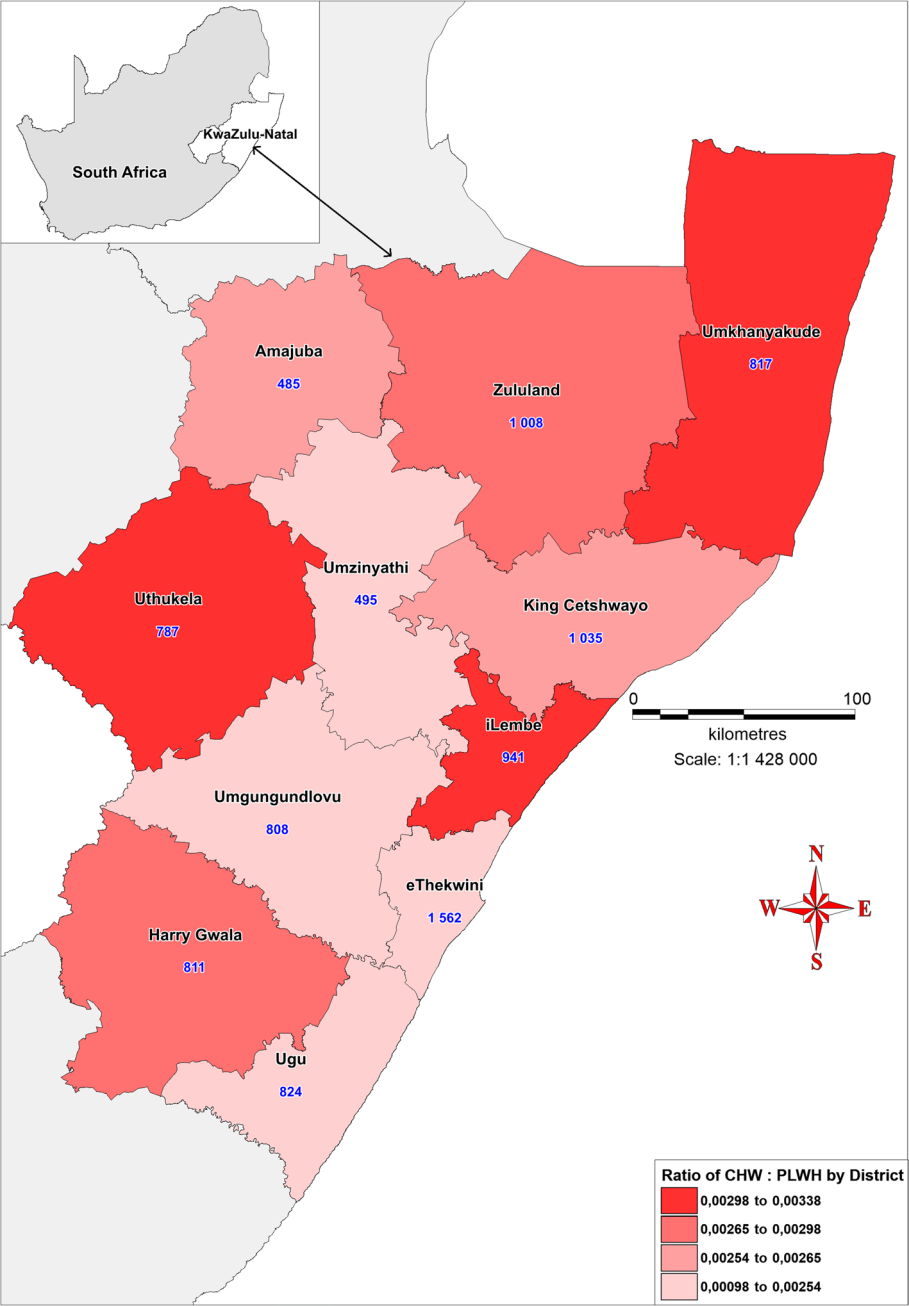
Choropleth Map showing the ratio of CHW to PLWH in the districts. The low intensity colour shows significant shortage of CHWs

As mentioned above, from the extrapolation of CHW: PLWH based on the KZN prevalence of 18.1%, a CHW:PLWH was calculated as 1: 109–181. As seen in Table 3., KZN has a CHW:PLWH ratio of 1: 290 which translate to shortage of CHWs in the districts. As seen in Fig. 3., only three districts, namely, iLembe, uThukela and uMkhanyakude met the estimated norm for the CHW: PLWH ratio, with ratios of 1: 137, 1: 154 and 1:154 respectively. While these three districts have varying HIV prevalences, they are the districts with the lowest numbers of PLWH which probably contributed to their higher CHW: PLWH ratio.

**Table 3:** Population, HIV prevalence, CHWs, PLWH, CHW:PLWH ratio and MPI scores in the KZN districts

KZN District	District Population^a^	HIV prevalence %^b, d^	No. of CHWs^c^	No. of PLWH^e^	CHW:PLWH Ratio	MPI Score^f^
**KZN Province**	**11,065,240**	**18.1%**	**9573**	**2,775,039**	**1: 290**	**0.05**
uMgungundlovu	1,095,865	46.6	808	276,224	1: 342	0.02
eThekwini	3,702,231	43.5	1562	964,800	1: 618	0.02
Ugu	753,336	43.4	824	253,859	1: 308	0.05
iLembe	657,612	43.1	941	129,154	1: 137	0.04
uMzinyathi	554,882	41.7	495	166,352	1: 336	0.07
uThukela	706,588	41.5	787	121,516	1: 154	0.04
Harry Gwala	510,865	39.2	811	155,234	1: 191	0.06
King Cetshwayo	971,135	39.1	1035	224,875	1: 217	0.03
Zululand	892,310	37.6	1008	211,562	1: 210	0.04
Amajuba	531,327	36.4	485	146,002	1: 301	0.02
uMkhanyakude	689,090	35.0	817	125,461	1: 154	0.07

^a^[*Source*: KwaZulu-Natal Community Survey, 2016 (Statistics, South Africa]

^b^[*Source*: The 2017 National Antenatal Sentinel HIV Survey, South Africa]

^c^[*Source*: KwaZulu-Natal Department of Health, Human Resource Electronic Persal system, 2019]

^d^[*Source*: The South African National HIV Prevalence, Incidence, Behaviour and Communication Survey, 2017]

^e^[Source: KZN-DOH DHIS data]

^f^[*Source*: Statistics South Africa: Provincial SAMPI scores, 2016]

**Fig. 4:**
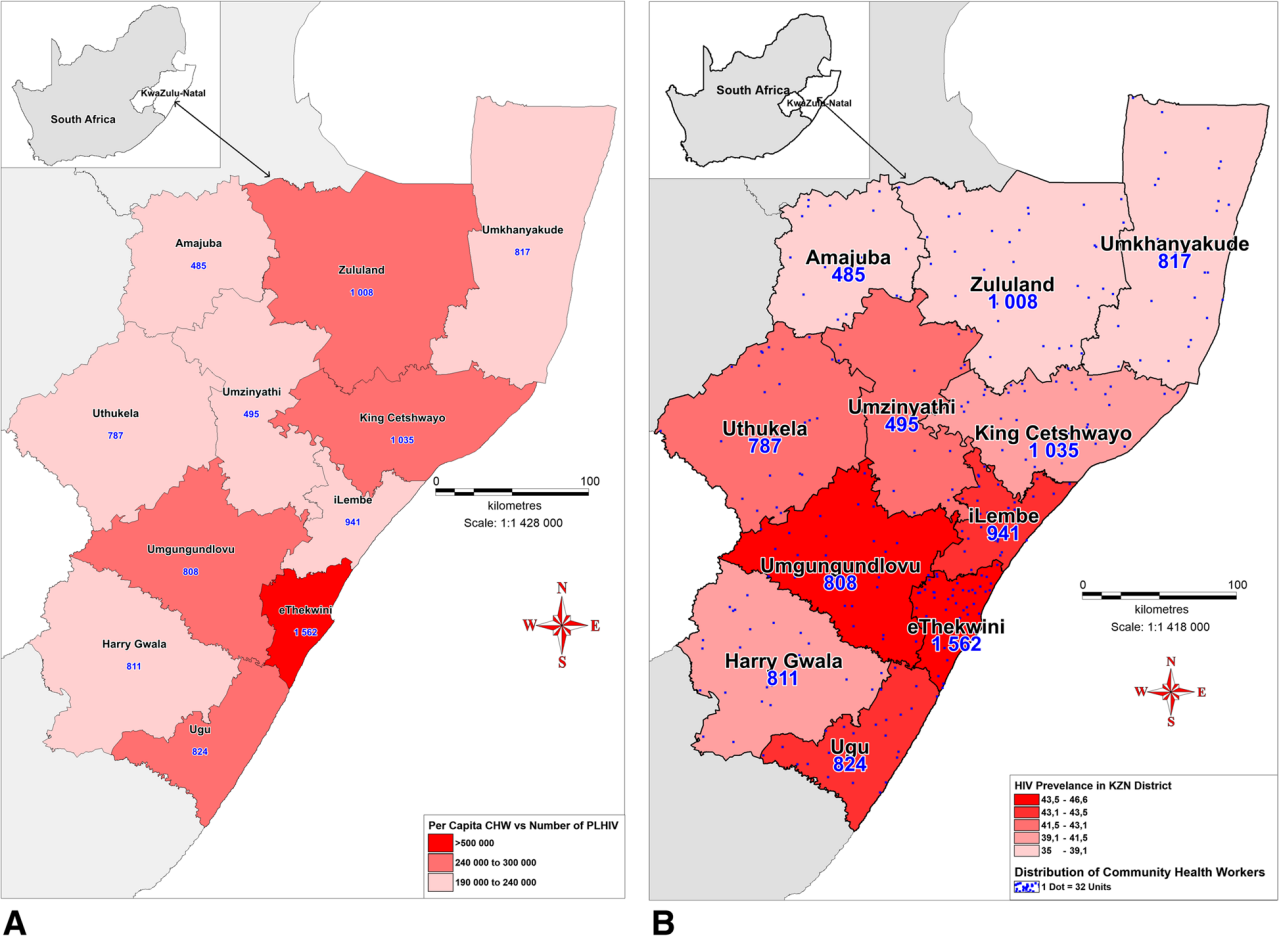
**A **Map showing the number of people living with HIV in KwaZulu-Natal districts (shaded areas) and per capita values of CHWs in each district (blue numerical values). The high intensity colour represents high numbers of people living with HIV. **B **Bivariate choropleth map showing spatial distribution of community health workers (dots) in relation to HIV prevalence (%) in KwaZulu-Natal districts. The high intensity colours representing districts with high HIV prevalence

As seen in Fig. 3., the two districts with the lowest CHW: PLWH ratio, indicative of a severe shortage of CHWs, were eThekwini and uMgungundlovu districts with ratios of 1: 618 and 1: 342 respectively. However, eThekwini district has a higher number (1562) of CHWs compared to uMgungundlovu district (808) which has the fourth lowest number of CHWs in the district. As seen in Fig. 4A, these two districts (and particularly eThekwini district) also have the highest HIV prevalence and highest numbers of PLWH, which may explain their low CHW: PLWH ratio. As seen in Fig. 4B., the intense colours of Ugu and iLembe districts indicate high HIV prevalence. However, unlike eThekwini, uMgungundlovu and Ugu, iLembe district met the estimated CHW: PLWH (1: 137) which was in fact the highest in the province.

UMkhanyakude and Amajuba districts have the first and second lowest HIV prevalences of 35 and 36.4% respectively. However, only uMkhanyakude district met the required CHW: PLWH ratio with a ratio of 1: 154 due to its higher numbers of CHWs (817) and lower numbers of PLWH (125461) compared to Amajuba. The low CHW: PLWH ratio in Amajuba district (1:301) is indicative of a shortage of CHWs. As seen in Table 3., Amajuba had the fewest CHWs (485) compared to other districts. Conversely, although King Cetshwayo district had the second highest number of CHWs in the province (1035), they also had a CHW: PLWH ratio (1: 217) that is indicative of a shortage of CHWs due to a high number of PLWH (224,875, which is the fourth highest in the province).

The misallocation of CHWs is worse in some districts than in others. Despite the differences in their population sizes, Harry Gwala and uMgungundlovu districts have distinct misallocation of CHWs based on their HIV prevalences as seen in Fig. 4B. Harry Gwala district has an HIV prevalence that is 7.4% lower than that of uMgungundlovu district. However, the two districts have almost an equal number of CHWs.

### Community health workers’ distribution as per poverty scores in the districts

**Fig. 5:**
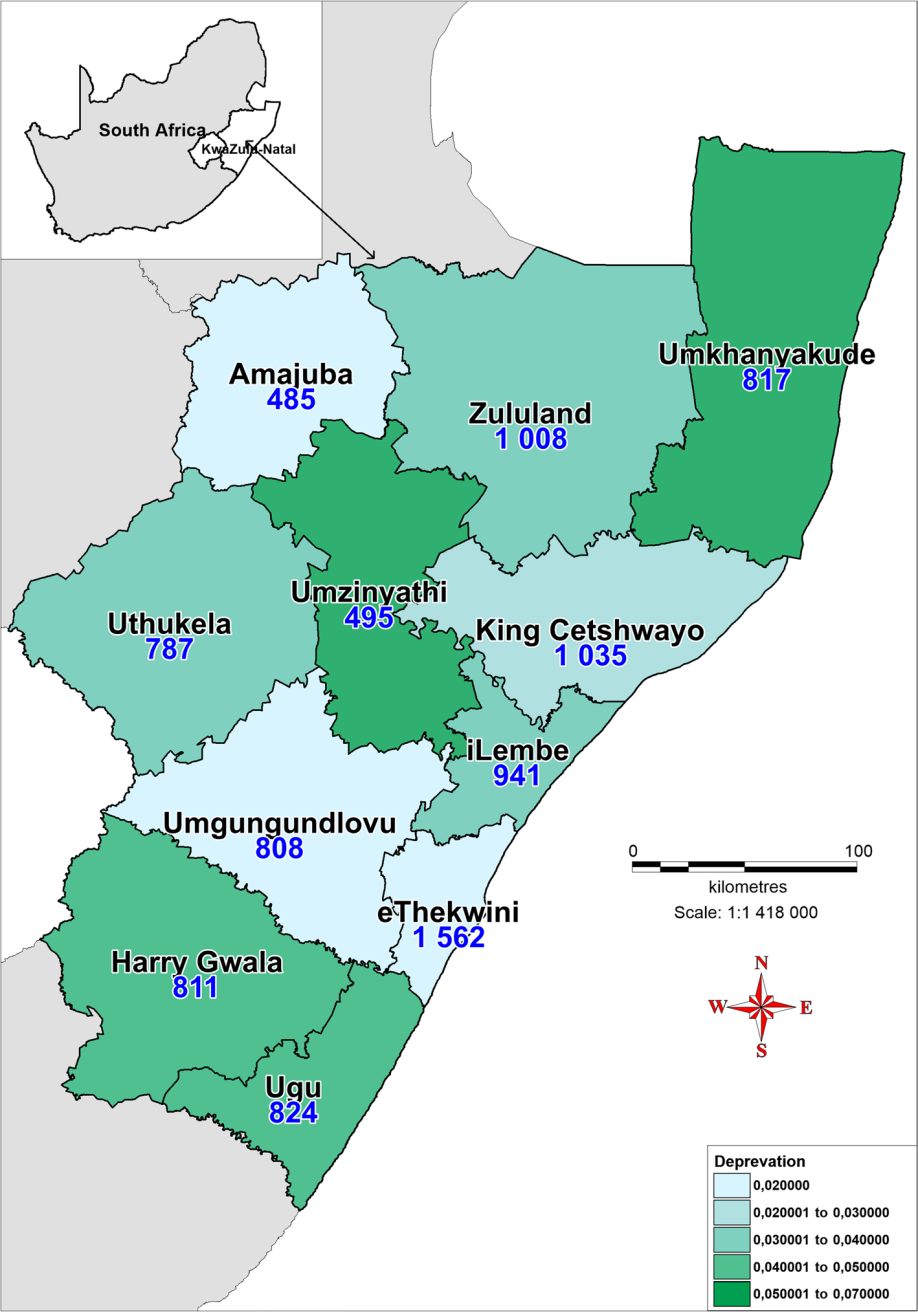
Choropleth map showing the poverty scores in the districts and per capita community health workers distributed in each district (black numerical values). The high intensity color shows areas that are most poor. The map was generated using MapInfo Pro 17.0 (https://www.filehorse.com/download-mapinfo-pro/)

As seen in Table 3, and Fig. 5, the richest districts with a MPI score of 0.02, namely, uMgungundlovu and eThekwini, not only have the same MPI score but are also the most populous in the province, and have the highest number PLWH, lowest CHW: people ratio and lowest CHW: PLWH. In comparison, although it has the same MPI score of 0.02, Amajuba district has the second lowest HIV prevalence, and the lowest number of CHWs and PLWH.

When looking at the poorest districts with an MPI score of 0.07, namely, uMzinyathi and uMkhanyakude districts, uMkhanyakude has the lowest HIV prevalence in the province but was allocated more CHWs than uMzinyathi district which had the fifth highest HIV prevalence. For this reason, uMkhanyakude met the CHW: PLWH ratio and uMzinyathi did not. Interestingly, it seems that the wealthier districts with a MPI score of 0.02, had a higher HIV prevalence. Overall, there was no clear pattern in the distribution of CHWs according to poverty lines as suggested in the policy framework**.**

### Comparison for all variables using ranking

Table 4. shows the ranking of the districts according to the following variables: district population; HIV prevalence; number of CHWs; number of PLWH; CHW: people ratio; CHW: PLWH ratio and MPI scores.

**Table 4:** Ranking within the variables

KZN Districts	District Population	HIV prevalence %	No. of CHWs	No. of PLWH	CHW: People Ratio	CHW:PLWH Ratio	MPI Score ^a^
uMgungundlovu	**2**	**1**	8	**2**	**10**	**10**	**1**
eThekwini	**1**	**2**	**1**	**1**	**11**	**11**	**1**
Ugu	5	3	5	3	6	8	8
iLembe	8	4	4	9	**2**	**1**	5
uMzinyathi	9	5	**10**	6	9	9	**10**
uThukela	6	6	9	**11**	5	**2**	5
Harry Gwala	**11**	7	7	7	**1**	4	9
King Cetshwayo	3	8	**2**	4	7	6	4
Zululand	4	9	3	5	4	5	5
Amajuba	**10**	**10**	**11**	8	8	7	**1**
uMkhanyakude	7	**11**	6	**10**	3	**2**	**10**

Largest value within variables ranked as 1

^a^wealthiest district/s ranked as 1

There is a distinct pattern observed whereby the most populous and wealthier districts, namely, eThekwini and uMgungundlovu district have the highest HIV prevalence, highest number of PLWH, lowest CHW: PLWH and lowest CHW: people ratio. These findings suggest a shortage of CHWs in these districts.

The opposite pattern is noted at Harry Gwala and iLembe, the two districts with the lowest CHW: people and CHW: PLWH ratios. Compared to eThekwini and uMgungundlovu districts, Harry Gwala and iLembe districts have lower HIV prevalences, lower numbers of PLWH, higher CHW:PLWH ratio (iLembe district has the highest in the province) and higher CHW: people ratio (Harry Gwala district has highest in the province). However, Harry Gwala still did not meet the estimated norm of CHW: PLWH (indicating a CHW shortage) because of the lower numbers of CHWs in the district. Additionally, the two districts did not rank the same for MPI scores with Harry Gwala district considered poorer (MPI of 0.06) than iLembe district (MPI of 0.04), ranking number 9 and number 5 respectively.

The above- mentioned patterns were not noticeable in all the districts. For example, the districts with the lowest HIV prevalence, namely Amajuba and uMkhanyakude, had varying CHW: people and CHW: PLWH ratios, where uMkhanyakude met the suggested norms for both CHW: people and CHW: PLWH ratios and Amajuba did not. This may be due to the fact that Amajuba had the lowest number of CHWs in the province and a slightly higher HIV prevalence than uMkhanyakude district. Furthermore, they had a significant MPI score difference with Amajuba ranking number as one of the richest districts and uMkhanyakude ranking as one of the poorest districts.

Overall, in most districts the CHWs did not seem to be allocated by population size, HIV prevalence, number of PLWH or poverty scores.

## Discussion

The study aimed at describing the distribution of CHWs in relation to the HIV prevalence in the KZN districts. Overall, KZN as a province did not meet the estimated allocation norms of CHWs, either with respect to the provinces’ general population or to the number of PLWH indicating an overall shortage of CHWs in the province. At district level, the results showed extensive geospatial heterogeneity with no obvious pattern in the distribution of CHWs not only in relation to the HIV prevalence but also in relation to the population size, number of PLWH and poverty scores. This is in line with the evidence which showed that there is disparity in the distribution of CHWs in KZN [22, 24] as well as wide variation of HIV prevalence between districts in the province [38, 44]. Furthermore, the study showed that although the majority of districts met the estimated allocation of CHWs in the district population, the majority of them suggested shortages of CHWs in terms of the CHW: PLWH ratio.

Despite the relatively high ratios of CHW: PLWH, the majority (seven) of the districts had an estimated CHW: people ratio that was within that extrapolated from the WBPHCOT policy framework (1: 600–1000) [23]. The provincial CHW: people ratio of 1: 1156, was higher than the estimated policy framework [23] ratio indicating an overall shortage of CHWs among the population of KZN. In general, KZN has a shortage of healthcare workers [45] and the CHWs are deployed to the districts to mitigate this shortage; this is difficult when CHWs themselves are also experiencing a shortage [46]. When considering the district populations, the districts with the highest populations were more likely to have shortages of CHWs as seen in uMgungundlovu and eThekwini districts. However, this does not mean that the least populous districts had appropriately allocated CHWs as seen in Amajuba district, which also had a shortage of CHWs.

This study also showed that the two districts with the highest HIV prevalence, had the greatest shortage of CHWs shown by low ratios of CHW: PLWH. However, this was not mirrored in other districts which also had a high HIV prevalence (as seen in iLembe district which is one of the districts with high HIV prevalence but had the highest CHW: PLWH ratio). Community health workers from eThekwini district have been reported to cover a five times higher population which has led to vocational exhaustion [46]. Interestingly, the only districts which showed appropriate and sufficient allocation of CHWs, namely iLembe, uThukela and uMkhanyakude district all met the suggested norm for CHW: people ratio indicative of a generally appropriate allocation of CHWs in these districts.

The suggested CHW: PLWH ratio of 1: 109–181, showed a shortage of CHWs in eight out of the 11 KZN districts, particularly at uMgungundlovu and eThekwini districts. If CHWs are under allocated in relation to PLWH, this may mean that the CHWs are not reaching the targeted individuals in the province. This study found that CHWs are more often under-allocated than over-allocated which indicates a gap in the CHW workforce deployed in the districts, as shown in previous studies conducted in KZN [26, 27]. Similarly, another KZN study showed that CHWs were not distributed according to the disease status of catchment populations [22].

With regards to poverty, the wealthier districts (namely uMgungundlovu and eThekwini) had higher HIV prevalences and as well as higher numbers of PLWH. This seems to contradict the studies that have shown associations between HIV infection and poverty [4–11]. However, Amajuba district, which is also one of the wealthier districts, had a low HIV prevalence. Furthermore, districts that were considered poor, for example uMzinyathi district, also had a low HIV prevalence**.** The four wealthiest districts with a MPI score of 0.02 and 0.03 and the two poorest districts with an MPI score of 0.07, showed no pattern in the district population, HIV prevalence, number of CHWs, number of PLWH, CHW: people ratio and CHW: PLWH ratio. The WBPHCOT policy framework suggests that CHWs should be allocated to the poor communities [23]. However, there was no clear pattern in the distribution of CHWs in relation to poverty scores in the districts. The results show inconsistencies in the allocation of CHWs in the poorest districts; for example, uMzinyathi district shows an extreme shortage of CHWs whereas uMkhanyakude district has an appropriate allocation of CHWs**.** Overall, the distribution of CHWs in the districts was neither predetermined by the poverty scores as stipulated in the WBPHCOT policy framework [23] nor followed a pattern according to the HIV prevalence observed in the districts. In contrast to prior studies [16–19], our study did not support the direct association of poverty and HIV. This may be because taking the district as a unit of analysis masked pockets of poverty within the districts where an association with HIV prevalence would have been seen. However, the association between poverty and HIV is multi-faceted and other factors should be taken into consideration when allocating resources according to poverty measurements [5].

What may confound the distribution of CHWs in KZN, is that the variations in the HIV prevalence are not only between districts but are also between the sub-districts within the districts [38]. The WBPHCOT policy framework states that the district health management must ensure an equitable spatial distribution of WBPHCOTs for the district population [23]. Furthermore, to optimize the CHW programme, the World Health Organization (WHO) suggests that the distribution of CHWs should be directed by the disease epidemiology, local geography, population density and anticipated demand for services [35, 36]. The rationale for the distribution of CHWs within the districts in KZN is unclear. The KZN population that is served by CHWs is mostly poor, lives in rural areas and is disproportionately affected by HIV [17–19, 21]. Spatial analysis for poverty showed different levels of CHW shortages in districts. However, when it comes to the distribution of CHWs, in addition to the MPI scores, the total population and the disease burden in the catchment population should be considered, as guided by the WBPHCOT policy framework.

Rural areas require 33% more CHWs than urban areas [47] which may account for the relative shortage of CHWs seen in the urban districts of our study. However, HIV prevalence should also be considered in CHW allocation. Areas of high HIV prevalence could indicate areas of high HIV transmission and therefore incidence [48]. These areas require intense community interventions that can be mobilized through the services delivered by the CHWs at households. Poverty levels must also be considered. Interestingly, a study conducted in Malawi found that the odds of HIV-positive status were 0.72 times lower in people from poorer households as compared to those from wealthier households (AOR = 0.72, 95% CI = 0.54–0.95) [49]. Malawi has reported an HIV prevalence of between 6.4 and 10% (which is much lower than in KZN) [50] and is classified as a low income country [51]. These contradictory findings may be caused by multiple factors which explain the different contexts in which HIV evolves in these two settings.

To our knowledge, this study is the first to be conducted in KZN, where the geo-located distribution of CHWs in relation to the HIV prevalence in the districts was described. This geospatial analysis study can assist in identifying under-resourced areas so that the necessary resources including human resources (CHWs) can be directed to these areas as means of curbing the HIV epidemic as in other African settings [44]. Furthermore, geospatial mapping holds a great potential for improving the efficiency of the HIV programme, for example, by targeting HIV testing or expanding access to HIV pre-exposure prophylaxis (PrEP) to areas with high HIV prevalences [18, 38]. Our study was limited by the arguable accuracy and quality of secondary data which may not reflect the true picture of the health problem. The accuracy of the DHIS data is debatable although it can be used as a guide in the absence of alternative data and to provide a broader picture. Nonetheless, the study findings can be used as a guide for directing interventions. In addition, using aggregated district data as opposed to disaggregated sub-district level data may also mask the true picture of the health problem. Data on CHW distribution was available at sub-district level but this was not the case for HIV prevalence data which was only available at district level. Notwithstanding, both South African provincial and district level HIV prevalence reports are from scientifically conducted research reports which are credible and reliable.

While our study considered the distribution of CHWs in relation to HIV prevalence, future geospatial analysis studies on CHW deployment in KZN, should consider multi-level approaches using other variables such as poverty stratification and other health determinants. Predisposition to HIV positivity is influenced by a wide range of economic, environmental, demographic, social, and behavioural factors [48]. Our study achieved its objective which was to describe the distribution of CHWs in relation to HIV prevalence in the districts of KZN.

## Conclusion

While there are some noticeable patterns in the allocation of CHWs in KZN, there is mostly extensive geospatial heterogeneity with no obvious explanation in the distribution of CHWs in relation to HIV prevalence, population size, numbers of PLWH and poverty levels in KZN. Furthermore, the inequality in the distribution of CHWs seen in this study may be associated with inequalities in the provision of HIV related services especially in districts with CHW shortages. Shortages of CHWs may result in missed opportunities in the provision of community-based HIV services for better health outcomes. These results provide motivation for a broad approach which will expand the CHW workforce, especially in districts with a high burden of disease and high levels of poverty as proposed in the WBPHCOT policy framework.

## Data Availability

The datasets used and/or analyzed during the current study are available from the corresponding author on reasonable request. The KZN-DoH is the custodian for the distribution of CHWs in the KZN districts data and KZN districts ‘shape files (available on request from KZN-DoH: http://www.kznhealth.gov.za). The KZN HIV prevalence data is publicly available on the *South African National HIV Prevalence, Incidence, Behaviour & Community Survey Report* (https://www.hsrcpress.ac.za/books/south-african-national-hiv-prevalence-incidence-behaviour-and-communication-survey-2017). The district HIV prevalence data is publicly available on the *South African National Antenatal Sentinel HIV Survey Report* (http://www.nicd.ac.za/wp-content/uploads/2019/07/Antenatal_survey-report_24July19.pdf). The number of PLWH in the KZN districts is available on the DHIS (https://kz.dhis.dhmis.org/dhis-web-commons/security/login.action) and accessed only by authorized users or by request from the KZN-DoH. The poverty data is publicly available on the South African Multidimensional Poverty Index Report (http://www.statssa.gov.za/publications/Report-03-10-08/Report-03-10-082014.pdf).

## Abbreviations

CHW: Community health worker
CHWs: Community health workers
GIS: Geographical Information System
HR: Human Resources
KZN: KwaZulu-Natal
KZN-DoH: KwaZulu-Natal Department of Health
HIV: Human immunodeficiency Virus
NDoH: South African Department of Health
OTL: outreach team leader
PHC: primary healthcare
PLWH: People living with HIV
Stats SA: Statistics South Africa
WBPHCOT: Ward Based Primary Health Care Outreach Team
WHO: World Health Organization
